# The Analgesic Efficacy of Therapies Used for Complex Regional Pain Syndrome: A Systematic Review

**DOI:** 10.7759/cureus.91697

**Published:** 2025-09-06

**Authors:** Charlotte Shekarsarai, Nicholas A McQuibban, Nicola Gullick

**Affiliations:** 1 Medicine, Queen Elizabeth Hospital, King’s Lynn, GBR; 2 Life Sciences, Imperial College London, London, GBR; 3 Rheumatology, University Hospitals Coventry & Warwickshire, Coventry, GBR; 4 Institute for Precision Diagnostics and Translational Medicine, University Hospitals Coventry & Warwickshire, Coventry, GBR; 5 Warwick Medical School, University of Warwick, Coventry, GBR

**Keywords:** analgesic effect, bisphosphonates therapy, complex regional pain syndrome stages, crps-1, crps-2, heath related quality of life, mirror therapy, non-pharmacological interventions, patient functional status, pharmacological interventions

## Abstract

Complex regional pain syndrome (CRPS) is a debilitating chronic pain condition that may develop after fractures, surgery, or soft tissue trauma. It is characterized by pain disproportionate to the initial injury, often accompanied by sensory, motor, autonomic, and trophic changes. Despite extensive research, pathophysiology remains unclear, and treatment approaches are varied, with inconsistent supporting evidence. Given its complexity and the potential for chronic disability, identifying effective therapies remains a clinical priority.

This review systematically evaluated the analgesic efficacy of pharmacological and non-pharmacological therapies for CRPS, based on randomized controlled trials (RCTs) published between 2003 and 2025. The protocol was developed previously and registered in PROSPERO (CRD420251026503).

A structured literature search using a PICO (Population, Intervention, Comparator, Outcome) framework was conducted across MEDLINE, PubMed, and the Cochrane Library. Search terms included “Complex Regional Pain Syndrome,” its earlier term “Reflex Sympathetic Dystrophy,” and intervention-specific keywords. Included were English-language RCTs in adults with clinically diagnosed CRPS (type I or II), assessing pain reduction as the primary outcome and function, quality of life, and pain medication use as secondary outcomes. Two reviewers independently screened and extracted studies. Risk of bias was assessed using the Cochrane Risk of Bias (ROB) 2. Publication bias was evaluated using funnel plots of standard errors and effect sizes.

In total, 45 RCTs met the inclusion criteria and included 2,125 patients. Among pharmacological interventions, bisphosphonates showed consistent and significant pain reduction over six months. Intravenous ketamine demonstrated strong short-term analgesia, though findings were limited by small samples, variable protocols, and lack of long-term data. Combinations of local anesthetics and other medications (e.g., lidocaine with citalopram or parecoxib) were especially effective in acute CRPS. Steroid treatments (oral or regional) offered short-term pain relief and functional improvement, particularly in early or post-stroke CRPS.

Non-pharmacological therapies also showed promise in reducing CRPS pain. Mirror therapy (MT) and graded motor imagery (GMI) consistently improved pain and motor function, especially when applied early. Pain exposure physical therapy (PEPT) improved range of motion but had a limited impact on overall functional outcomes. Neuromodulation methods, including spinal cord stimulation (SCS), dorsal root ganglion (DRG) stimulation, and transcutaneous electrical nerve stimulation (TENS), provided durable pain relief in select patients but were technically complex and associated with complications, particularly with SCS.

In conclusion, CRPS remains a complex and difficult-to-treat condition, with substantial variability in treatment response. RCT evidence supports the use of bisphosphonates, ketamine, and early use of mirror or motor imagery therapies. Neuromodulation via electrical stimulation may benefit select cases but carries procedural risks. Physiotherapeutic strategies offer low-cost, low-risk benefits, especially when started early or combined with pharmacotherapy. However, many studies were limited by small size, short follow-up, or methodological flaws. There is an urgent need for large, high-quality, and mechanistically informed RCTs to guide long-term CRPS management.

## Introduction and background

Complex regional pain syndrome (CRPS) is a chronic neuropathic pain disorder typically caused by commonplace trauma, characterised by continuous burning pain, allodynia, hyperalgesia, and autonomic dysfunction that is disproportionate to the inciting event and involving multifactorial pathophysiology including inflammatory, immune, and central sensitisation mechanisms [1,2]. Current treatment relies on physical therapy and pain relief, but there are no universally accepted interventions [2]. This is partly due to gaps in knowledge, unclear pathophysiology, patient variability in treatment responses, issues with classification, and conflicting findings for each intervention.

The overall incidence of CRPS is 26.2 per 100,000, with the highest incidence in postmenopausal females between the ages of 60 and 69, thought to be due to increased prevalence of osteoporosis and fractures [1]. The underlying pathophysiology is thought to include an element of autoimmunity, maladaptive neuroplasticity, central/peripheral sensitization, and excess inflammation. Clinically, CRPS can present in acute and chronic phases, with chronicity defined by symptoms being present for longer than six months. With time, a change from warm to cold CRPS is often reported, where patients progress from a red, burning limb to a cold, blueish limb with reduced motor function [2]. Furthermore, CRPS can be divided into types 1 and 2, depending on whether a nerve lesion is present or not, respectively. Diagnosis of CRPS has proven to be challenging; the original diagnostic criteria were established by the International Association for the Study of Pain (IASP) in 1994 [3], but concerns over sensitivity and specificity led to revision. In 2003, the Budapest Clinical Criteria were proposed for diagnosis and subsequently validated in 2010 [4]. These criteria require a minimum of three symptoms, such as allodynia and temperature sensitivity, and two clinical signs, such as erythema and oedema [3,4].

This review aims to discuss existing interventions investigated for analgesic effect in patients with CRPS.

## Review

Methods

Objectives, Registration, and Protocol

The latest Preferred Reporting Items for Systematic Reviews and Meta-Analyses (PRISMA) 2020 framework was used to structure the literature search, abstract and full text screening, and enforcement of inclusion and exclusion criteria [5]. A PICOS (participants/population, interventions, comparisons, outcomes, and study design) framework was developed [6] to assess the efficacy of both pharmacological and non-pharmacological interventions for pain due to CRPS, as seen in Table 1.

**Table 1:** PICO criteria for the systematic review PCOS, participants, intervention, comparison, outcome

PICO criteria	Criteria description
Patient	Adult (age 18 years and older) subjects with either acute or chronic CRPS, regardless of subtype (CRPS type I or II).
Intervention	Pharmacological and non-pharmacological therapies aimed at pain relief (e.g., bisphosphonates, ketamine, mirror therapy, motor imagery, pain exposure physical therapy, steroids, and neuromodulation).
Comparison	Placebo, standard care, no treatment, or alternative therapeutic interventions.
Outcomes	Primary: pain reduction; Secondary: motor function improvement, range of motion, overall impairment scores, durability of effect, adverse events.

Study Criteria

The protocol was developed a priori and registered in PROSPERO (CRD420251026503) [7]. Specific inclusion and exclusion criteria are shown in Table 2.

**Table 2:** Inclusion and exclusion criteria for the systematic review IASP, International Association for the Study of Pain

Inclusion criteria	Exclusion criteria
English language	Studies in other languages
Randomized controlled trials	Cohort studies, case reports, case studies
Adult population (≥18 years)	Adolescents (under 18 years of age)
Clinically diagnosed complex regional pain syndrome (as per the Budapest/IASP criteria)	
Studies between the years 2003 and 2025	

Information Sources

The following databases were searched from 2003 to 2025: MEDLINE, PubMed, and COCHRANE LIBRARY.

Search Strategy

A comprehensive literature search was conducted across MEDLINE, PubMed, and the Cochrane Library to identify relevant reviews and randomized controlled trials (RCT). The search was restricted to articles published in English. Keywords and MeSH terms included “Complex Regional Pain Syndrome”, as well as its historical synonyms such as “Reflex Sympathetic Dystrophy” and “Sudeck’s Atrophy.” These were combined using Boolean operators with terms related to intervention types, including “drug therapy,” “pharmacological treatment,” “physiotherapy,” “physical therapy modalities,” “psychological therapy,” “cognitive behavioral therapy,” “interventional therapy,” “nerve block,” and “neuromodulation.” The final search string incorporated filters for study design, specifically “randomised controlled trial” and “RCT,” to ensure methodological rigor. Filters were applied to ensure database searches remained between 2003 and 2025 only. Reference lists of included studies were also screened to identify any additional eligible trials not captured through database searches.

Selection of Studies and Extraction of Data

Abstract screening was performed by two separate authors (CS and NG) through the screening tool Rayyan (https://rayyan.ai) [8], and all discrepancies were resolved through discussion. Full text screening was then completed by two separate authors (CS and NM), using the same tool. Data was extracted by two separate authors (CS and NM) using a data extraction form, which was first piloted on four studies. The form included study characteristics, participant demographics, details of intervention and comparator, and outcome measures of interest.

Data Synthesis, Assessment of the Risk of Bias, and Critical Appraisal

This review assessed the methodological quality of included RCTs using the Cochrane Risk of Bias 2 (RoB 2) tool [9]. This tool evaluates bias across five domains: randomization process, deviations from intended interventions, missing outcome data, measurement of the outcome, and selection of the reported result. Two reviewers (CS and NM) independently assessed each study, with disagreements resolved through discussion. Risk of bias judgments (low risk, some concerns, or high risk) were recorded for each domain and used to inform the overall study-level risk of bias; these were summarized in a risk of bias table and figure, with visualizations created using the robvis web tool [10]. Overall publication bias, based on primary outcome results, was also assessed via a funnel plot to highlight any potential asymmetry across the selected studies.

A meta-analysis was planned where the data for the same outcome were available for at least three studies; if not, a narrative synthesis would be performed. Two subgroup analyses, by CRPS acuity (acute vs. chronic) and CRPS type (type 1 vs. type 2), were pre-specified to explore potential differences in treatment effects; however, it was anticipated that these analyses may not be feasible due to the likely limited representation of acute and type 2 CRPS.

Results

Study Selection and PRISMA Flow Diagram

**Figure 1:**
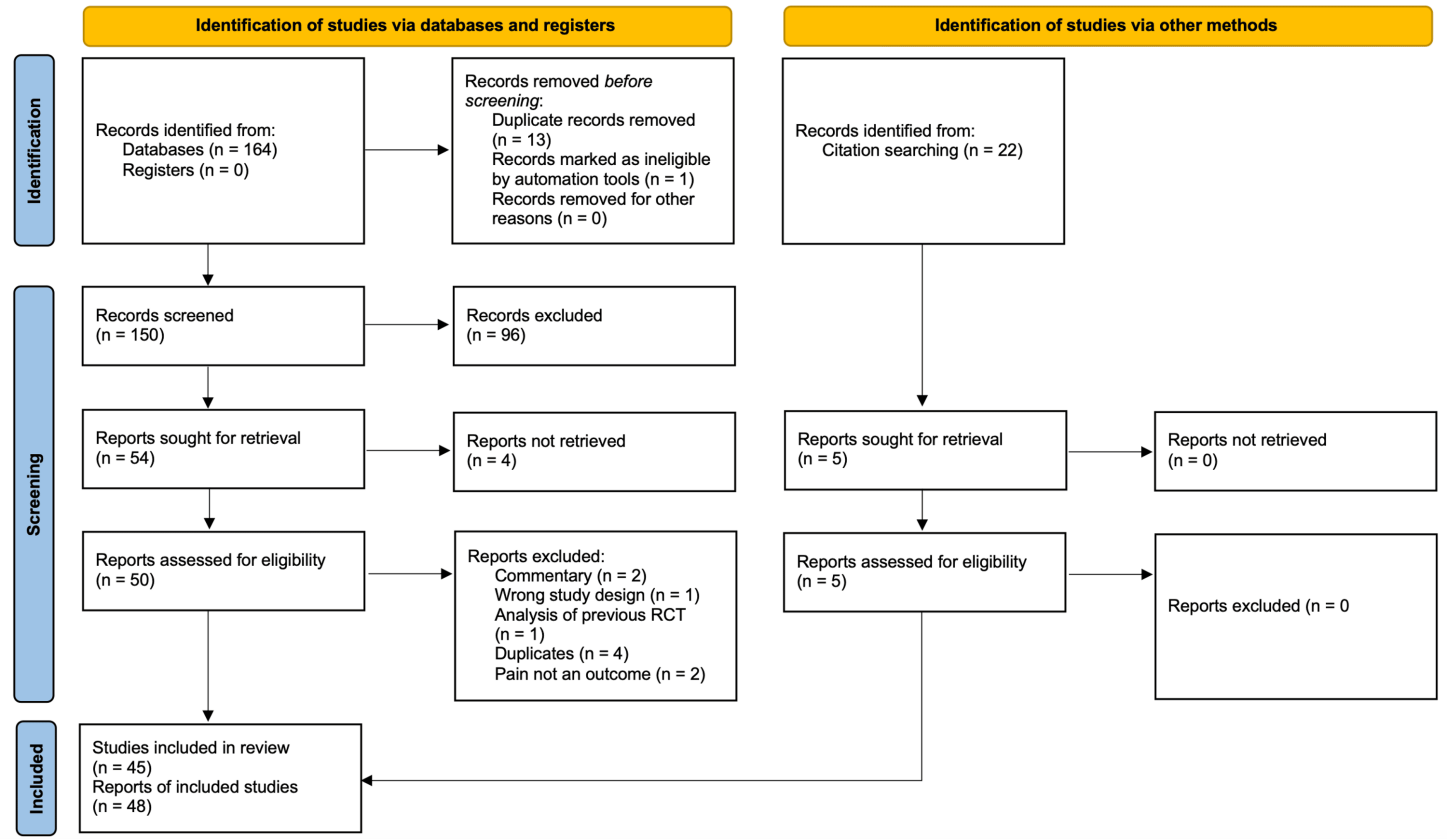
PRISMA flow diagram depicting the screening processes used from database searching and citation searching PRISMA, Preferred Reporting Items for Systematic Reviews and Meta-Analysis

Overall, 164 studies were identified using database searches. Following the screening processes, 53 reports were included, belonging to 45 unique studies. The rest were excluded due to study designs other than RCTs and outcomes other than pain, function, quality of life, and/or need for rescue medication. The diagram below (Figure 1) highlights this full process.

Risk of Bias Assessment and Publication Bias

**Figure 2:**
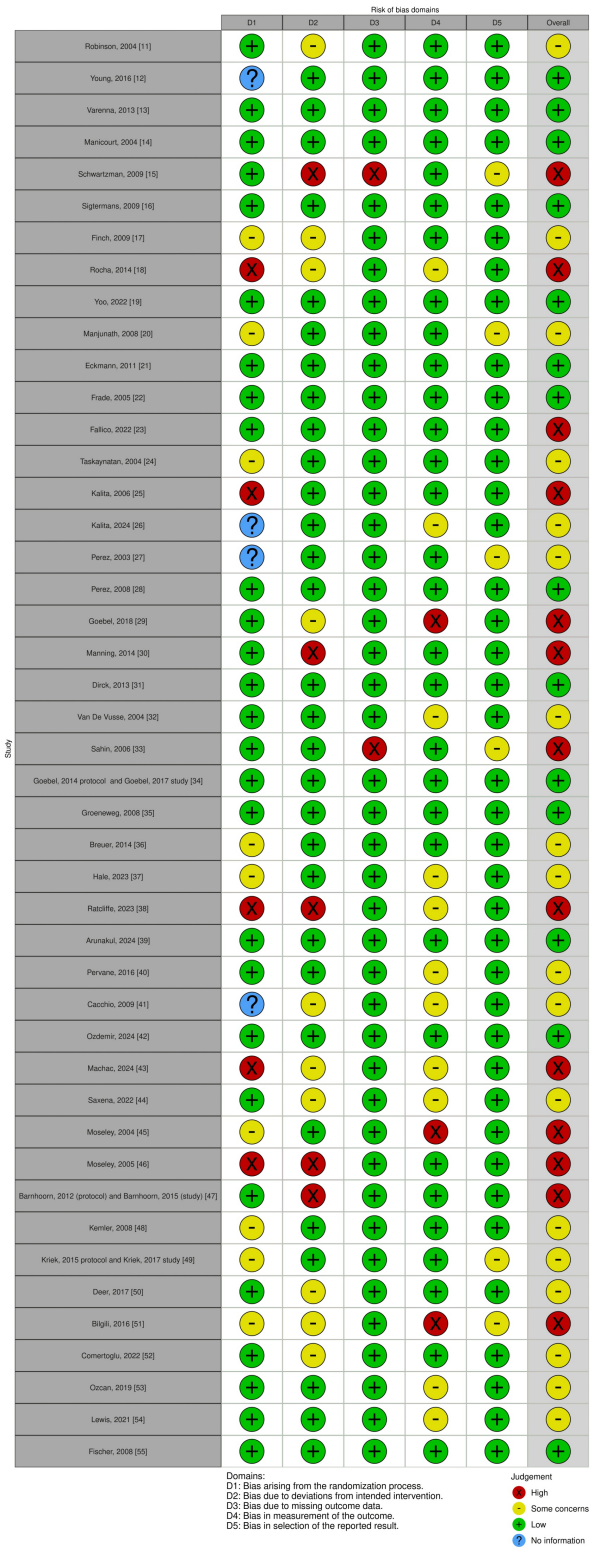
Individual RoB-2 scores across the five domains, as well as the overall score, for each publication ROB, risk of bias

**Figure 3:**
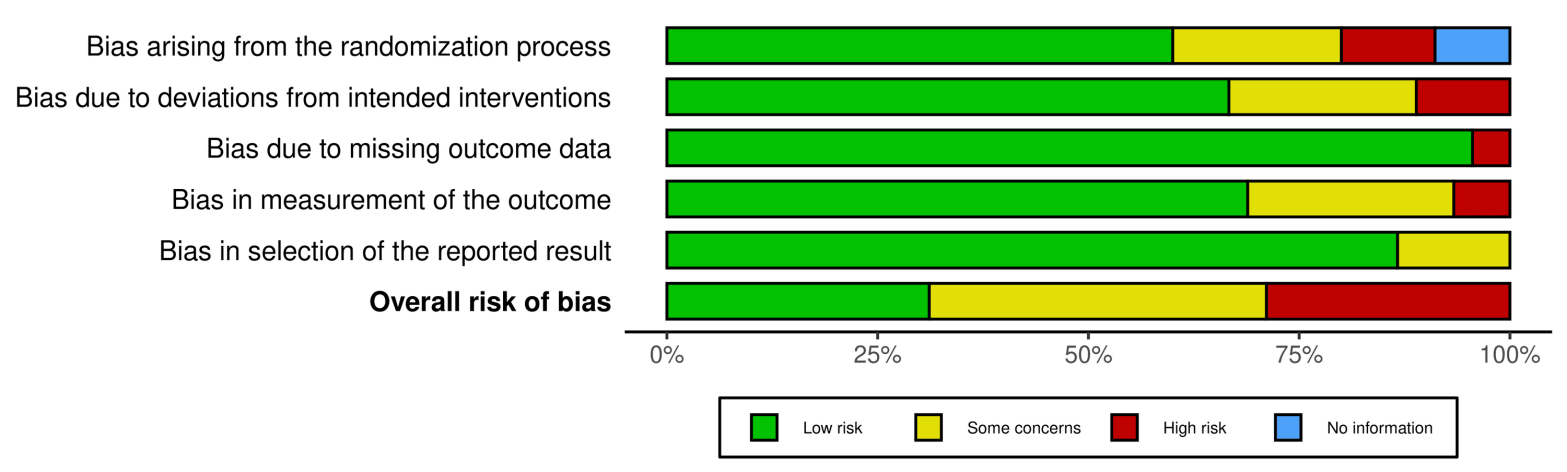
RoB-2 summary for all studies included in this systematic review ROB, risk of bias

Each included RCT was independently assessed for risk of bias using the Cochrane RoB-2 tool, which evaluates bias across five domains: randomization process, deviations from intended interventions, missing outcome data, measurement of the outcome, and selection of the reported result. Two reviewers conducted the assessments, with discrepancies resolved by discussion, ensuring a rigorous and standardized evaluation of study quality. RoB-2 scoring for each RCT was visualized using the robvis open-source web tool, as seen in Figure 2. Of the included RCTs, 31.8% had low risk of bias, indicating strong methodological quality. About 40.9% showed some concerns, suggesting minor issues that warrant cautious interpretation. Meanwhile, 27.3% were at high risk of bias, reflecting significant flaws that could affect the reliability of the result. These summary statistics are shown in Figure 3, highlighting varying study quality and the need to interpret findings carefully.

**Figure 4:**
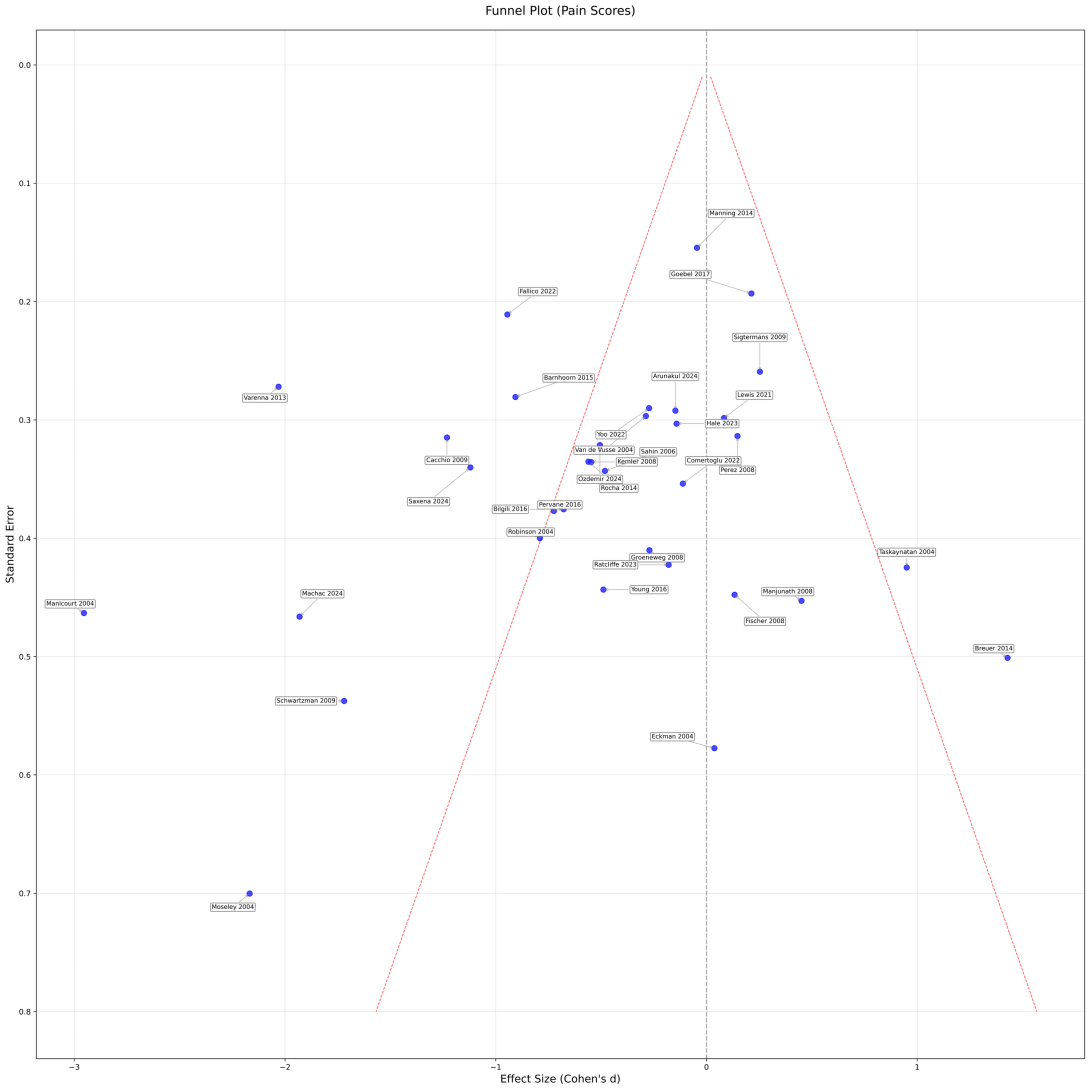
Funnel plot of all studies with pain scoring (e.g., visual analog scale and neuropathic pain scale): effect sizes versus standard error NB: some studies were excluded due to missing data and/or invalid primary outcome measurements

To assess potential publication bias related to the primary outcomes, a funnel plot was constructed plotting the effect sizes (Cohen’s d) against their standard errors. Visual inspection of the funnel plot allowed identification of asymmetry that might suggest the presence of publication bias, with more precise studies expected to cluster near the average effect and smaller studies symmetrically distributed around this mean, as shown in Figure 4. This combined approach provided a comprehensive appraisal of the internal validity and potential reporting biases within the included literature.

Findings on Pain Reduction for Pharmacological Interventions

All pharmacological interventions measured pain reduction as a primary outcome. Table 3 (bisphosphonates), Table 4 (ketamine), Table 5 (sympathetic blocks), Table 6 (local anesthetic), and Table 7 (“other mechanisms,” table for unique/novel methods) show pharmacological interventions used, with each table grouped by mechanism of action, along with CRPS subtype, study characteristics (demographics and size of patient populations), primary and secondary outcomes, and study results.

**Table 3:** Bisphosphonates studies and findings IV, intravenous; CRPS, complex regional pain syndrome; VAS, visual analog scale; SF-36, 36-item short-form health survey; NSAID, nonsteroidal anti-inflammatory drug

Author, year	Diagnosis	Intervention	Patient groups	Demographics	Primary and secondary outcomes	Results
Robinson, 2004 [11]	CRPS-1, chronic	IV pamidronate 60 mg single infusion vs. normal saline infusion. Background analgesia continued.	27 total: 13 lower limb, 14 upper limb; 13 placebo, 14 treatment	Mean age: 45; Male: 33.3%	Pain scores using Visual Analog Scale (VAS). Patient’s global assessment of disease severity documented at baseline, 1 month, and 3 months.	Average disease severity score was significantly lower in the treatment group at 3 months (7.6 to 5.3) vs. the control (5.8 to 5.3) (P=0.043). Percentage change in VAS was also significantly greater in the treatment group (P=0.048) at 3 months. Overall patient function using SF-36 was significantly better at 3 months (P=0.047).
Young, 2016 [12]	CRPS-1, chronic	60 mg pamidronate mixed with 500 mL saline, IV infusion over 4 hours vs. oral prednisolone 1 mg/kg, dose tapered over 14 days.	21 total: 10 steroids, 11 pamidronate	Mean age: 65; Male: 50%	Pain improvement (VAS scores) at 1, 2, and 4 weeks.	Pamidronate was as effective as prednisolone for significant pain relief at weeks 1, 2, and 4 (P<0.01). No significant time-by-group interactions were found (4.60±0.84 in steroid vs. 4.00±1.90 in pamidronate) (P=0.205).
Varenna, 2013 [13]	CRPS-1, upper and lower extremity, chronic	IV infusion of 100 mg neridronate given four times over 10 days, every third day vs. placebo. Both diluted in 500 mL isotonic saline solution and infused in the morning (2 hrs).	82 total: 41 per group	Mean age: 57.5; Male: 35%	VAS 40 days after the first infusion. Secondary: SF-36, number of NSAIDs taken weekly.	A 50% decline in VAS score was reported in 73.2% of treated patients compared to 32.5% of controls (P=0.0003) 40 days post-infusion. SF-36 scores showed significant reductions in all components except mental. All patients on neridronate and 45% on placebo stopped using NSAIDs within 2 weeks.
Manicourt, 2004 [14]	CRPS-1, lower limb, chronic	Oral alendronate 40 mg	10 placebo, 10 alendronate	Mean age: 45; Male: 48%	VAS score at weeks 4, 8, 12 (0-100 scale).	Treatment group: VAS significantly reduced by week 4 (P<0.05). Placebo: VAS reduced by week 12 (P<0.05). The mean VAS score in the alendronate-treated group was 33% of that in the placebo-treated group.

**Table 4:** Ketamine studies and findings CRPS, complex regional pain syndrome; IV, intravenous; VAS, visual analog scale; AROM, active range of motion; PLO, pluronic lecithin organogel

Author, year	Diagnosis	Intervention	Patient Groups	Demographics	Primary and secondary outcomes	Results
Schwartzman, 2009 [15]	CRPS, chronic, failed 3 therapies	10-day ketamine IV infusion with 100 mL of normal saline vs. placebo saline without ketamine. Infused 4 h daily (25 mL/h) for 10 days.	21 total: 9 placebo, 10 treated; 2 dropouts	Mean age: 42; Male: 5%	Thermal pain, allodynia, deep pressure pain, motor testing. Measured at 2 weeks and 3 months.	Study stopped halfway through. Over 2 years, doses changed from 25 mg to 50 mg, deemed safe and more effective.
Sigtermans, 2009 [16]	CRPS-1, chronic	Ketamine IV infusion rate started at 1.2 μg/kg/min (or 5 mg/h for a 70-kg patient) at 8 AM on day 1 and was titrated at regular intervals (max. thrice daily) to a maximum of 7.2 μg/kg/min (or 30 mg/h for a 70-kg patient).	60 total: 30 per group	Mean age: 45; Male: 20%	Pain score - VAS over 12 weeks. Secondary: activity, walking ability, AROM, allodynia, temperature, and edema.	At 3 months, significance in pain relief between groups was lost (P=0.07). No functional improvement reported.
Finch, 2009 [17]	18 CRPS-1, 2 CRPS-2; 12 upper limb, 8 lower limb; chronic	Topical ketamine PLO, a microemulsion-based gel vs. placebo PLO vehicle without ketamine.	20 adults: 10 per group	Mean age: NA; Male: 30%	Sensory disturbance: light touch, pressure, punctate stimulation, light brushing, and thermal stimuli, measured 30 min after 10% ketamine cream application.	Pain did not improve following the use of ketamine cream or a placebo. Allodynia was significantly reduced with ketamine cream (P<0.01) (60% allodynia present to ~15% after cream).

**Table 5:** Sympathetic block studies and findings CRPS, complex regional pain syndrome; TSB, thoracic sympathetic block; VAS, visual analog scale; BPI, brief pain inventory; WHOQOL-BREF, World Health Organization Quality of Life – Brief version; RF, radiofrequency; NRS, numerical rating scale

Author, year	Diagnosis	Intervention	Patient groups	Demographics	Primary and secondary outcomes	Results
Rocha, 2014 [18]	CRPS-1, chronic	TSB group: 10 mL of anesthetic + corticosteroid solution (5 mL of 0.75% ropivacaine + 5 mL of 2% triamcinolone) injected into the T2 sympathetic thoracic ganglion. Control: 10 mL of anesthetic + corticosteroid solution (5 mL of 0.75% ropivacaine + 5 mL of 2% triamcinolone) injected into the subcutaneous space.	37 total: 18 TSB, 19 control	Mean age: 43; Male: 0%	Average pain score using BPI measured at 4 weeks; quality of life measured using WHOQOL-BREF at 12 months.	At 1 month, no statistically significant differences were reported between groups. At 12 months, the average pain item was significantly lower in the TSB group (3.47±3.5) compared to the control group (5.86±2.9; P=0.046). Quality of life was only slightly improved by TSB.
Yoo, 2022 [19]	CRPS-1, CRPS-2, lower limb, chronic	Treatment group: blocking injections containing 8 mL of 0.25% levobupivacaine and botulinum toxin type A 75 IU at L2. Control: saline solution injection at the same site.	48 total: 24 per group	Mean age: 43.5; Male: 50%	Temperature changes (not assessed in this review); pain intensity using an 11-point NRS.	Pain intensity was greatly reduced in the botulinum toxin group compared with the control group at 1 month (–2.2±1.0 vs. -1.0±1.6, P=0.003) and 3 months (–2.0±1.0 vs. –0.6±1.6, P=0.003).
Manjunath, 2008 [20]	CRPS-1, lower limb, chronic	Percutaneous RF lumbar sympathectomy vs. 7% phenol lumbar sympathectomy. No placebo or sham.	20 total: 10 per group	Mean age: 38.9 in RF, 51.6 in phenol. Male: NA	Pain outcomes using 9 measurement scales: VAS score, intensity of pain, sharp pain, hot pain, dull pain, sensitive sensation, unpleasant sensation, deep pain, and surface pain, each scored 0-10 at baseline.	Both procedures were safe and effective, providing significant pain relief (approximately 55% decrease in VAS scores) (P<0.05). No significant differences were found between the two treatments.

**Table 6:** Local anaesthetics studies and findings CRPS, complex regional pain syndrome; IV, intravenous; NRS, numeric rating scale; IVRAPG, intravenous regional anesthesia with parecoxib group; SPG, systemic parecoxib group; CG, control group; ISS, impairment level sum score

Author, year	Diagnosis	Intervention	Patient groups	Demographics	Primary and secondary outcomes	Results
Eckmann, 2011 [21]	CRPS-1, lower extremity, chronic >10 months	Four blocks performed over 4 weeks, in a random order of ketorolac doses (0, 30, 60, 120 mg). Each performed 1 week apart with 50 mL lidocaine of 0.5%.	12 total: 6 per group	Mean age: 37.9; Male: 50%	Overall pain NRS (0-10) at 1 week; pain with motion.	One day of significant pain reduction in the ketorolac groups, greatest at 30 mg and 120 mg (median NRS 6 to 4) (P=0.002). Pain with movement improved 7.15±0.69, 5.7±1.07, 6.1±0.86, 5.0±0.97, and 5.6±0.86 at baseline, 0, 30, 60, and 120 mg, respectively (P=0.059).
Frade, 2005 [22]	CRPS-1, upper extremity, chronic 7-18 months	IVRAPG: 5 mg parecoxib regionally, clonidine, and lidocaine. SPG: 20 mg parecoxib diluted in 0.9% physiologic solution systemically, clonidine, and lidocaine. CG: IV saline regionally.	30 total: 10 per group	Mean age: 41; Male: 66%	Analgesic efficacy: mean daily rescue analgesic consumption of 100 mg ketoprofen tablets over the following week (up to 3 tablets per day) + VAS weekly.	IVRAPG showed a 65% decrease in ketoprofen consumption between weeks 1 and 2 (P<0.05). VAS scores were similar during the first and second week observation, but IVRAPG showed smaller VAS scores in the third week (3.1 to 0.6) compared with both CG and SPG (P<0.05).
Fallico, 2022 [23]	CRPS, upper extremity, acute and chronic	Group 1: Lidocaine 5 cc every 10 days + citalopram oral drops 40 mg/mL, 7, 10, 15 drops for 1 year. Group 2: Lidocaine 5 cc every 10 days + placebo drops. Group 3: Placebo injections + placebo drops.	150 total: G1 acute 28, chronic 22; G2 acute 31, chronic 19; G3 acute 32, chronic 18	Mean age: 56.8; Male: 37%	ISS; decreased pain and swelling, increased range of motion.	Group 1: 73.5% reduction. Group 2: 54.7% reduction. Group 3: 36.6% reduction (P<0.0001). Subanalysis: acute > chronic; 1 month - significant reduction; 12 months - extremely significant.

**Table 7:** Other pharmacological studies and findings CRPS: complex regional pain syndrome; VAS: visual analog scale; NRS: numerical rating scale; BPI: brief pain inventory; SF-36: short-form (36) health survey; DSIS: daily sleep interference scale; ISS: impairment level sum score; HADS: hospital anxiety and depression scale; PGIC: patient global impression of change; QoL: quality of life; IV: intravenous; IVIg: intravenous immunoglobulin; DMSO: dimethyl sulfoxide; NAC: N-acetylcysteine; NaCl: sodium chloride; PDE-5: phosphodiesterase type 5; TDS: three times daily; BID: twice daily; TID: three times daily; BI: Barthel index; N_2_O, nitrous oxide; PROMIS-29, Patient-Reported Outcomes Measurement Information System 29-Item Profile

Author, year	Diagnosis	Intervention	Patient groups	Demographics	Primary and secondary outcomes	Results
Taskaynatan, 2004 [24]	CRPS-1, upper extremity, acute or chronic, not specified	Bier block using 40 mg methylprednisolone + 10 mL of 2% lidocaine vs. control 100 mL saline	25 total: 14 treatment, 11 placebo	Mean age: 22.3; Male: 100%	VAS pain 0-10, once a week for 3 weeks, 1 hour after treatment. Secondary: range of motion	During the 3 weeks, improvement in pain severity was statistically significant (P<0.05 in both groups); however, this was no longer present at the 1-month check-in
Kalita, 2006 [25]	CRPS-1, upper extremity, acute or chronic, not specified	Oral prednisolone 40 mg/day for 14 days, followed by 10 mg/week taper. Control group received oral piroxicam 20 mg/day	60 total: 30 per group	Mean age: 56; Male: 67%	Improvement in CRPS score (>2 points) at 1 month (aggregate of pain, edema, passive range of motion). Secondary: improvement in BI	Steroid group: score reduced from 10.73±1.95 to 4.27±2.83 (P=0.0001). Piroxicam group: score reduced from 9.83±2.34 to 9.37±2.89, not significant (P=0.24). BI score higher but not significant (P=0.06)
Kalita, 2024 [26]	CRPS-1, upper extremity, chronic	Prednisolone 40 mg or 20 mg via random allocation for 2 weeks, tapering over the next 2 weeks. Blood glucose monitored twice weekly; fasting and postprandial levels recorded. Follow-up at 1 month for VAS, CRPS score, and cytokine profiling	39 total: 20 mg group 19, 40 mg group 20	Mean age: 52; Male: 59%	Primary: pain improvement (VAS) at 1 month. Secondary: improvement in DSIS, total CRPS score	All patients showed >50% improvement in VAS score; seven patients reached VAS 0. Significant improvement in total CRPS score (P<0.001) and DSIS (P<0.001). Improvement comparable between the 20 mg and 40 mg groups. TNF-α decreased (P=0.046), IL-10 increased (P=0.018); TNF-α remained elevated, IL-10 remained low compared to controls
Perez, 2003 [27]	CRPS-1, limited to one extremity, acute and chronic (<1 year)	50% DMSO cream five times daily vs. NAC 600 mg effervescent tablets three times daily, both combined with placebo for 17 and 52 weeks	145 total: 74 NAC, 71 DMSO	Mean age: 49.5; Male: 33.8%	ISS 5-50, VAS, McGill pain questionnaire, active range of motion	No significant differences between NAC and DMSO at 17 or 52 weeks. Warm-type CRPS-1: DMSO more favorable; cold-type CRPS-1: NAC more effective
Perez, 2008 [28]	CRPS-1, upper and lower extremities, acute and chronic	Mannitol IV in 1 L 0.9% NaCl over 4 hours for 5 consecutive days vs. equal volume 0.9% NaCl placebo	41 total: 19 placebo, 22 mannitol	Mean age: 45; Male: 19.5%	VAS scores over 9 weeks post-treatment. Hand function using the Jebson Taylor test	No significant differences between mannitol and placebo for weeks 1-9 in maximum and mean VAS. Only the “stacking checkers” part of the Jebson Taylor test showed improved function (P<0.05)
Goebel, 2018 [29]	CRPS-1, upper extremity, chronic (>2 years)	Randomized 1:1 to mycophenolate as add-on vs. usual treatment over 5.5 months	12 total: 4 active, 5 control	Mean age: 43; Male: 0%	Average pain intensity over last 2 weeks (NRS 0-10). Secondary: QoL (EQ-5D) and function (BPI interference scale)	Two patients had >50% pain relief, one >40%, one >25%; these four also had improved secondary outcomes (P=0.01). Control: no patient >10% pain relief
Manning, 2014 [30]	CRPS-1, upper and lower extremities, chronic	Oral lenalidomide 10 mg once daily vs. placebo; concomitant analgesia allowed if stable for 1 month; no new CRPS therapy. 12 weeks total	184 total: placebo 94, lenalidomide 90	Mean age: 44; Male: 20%; 93% white, 4% black, 2% Asian/Hispanic	Reduced pain in index limb (>30% improvement, 11-point NRS). Secondary: activity and allodynia	Endpoint not met; equal proportions of treated (16.1%) and control (16.1%) achieved outcomes
Dirck, 2013 [31]	CRPS, upper and lower extremities, acute and chronic	Infliximab 5 mg/kg IV at weeks 0, 2, 6 vs. placebo + physiotherapy once/week	13 total: 6 treatment, 7 control	Mean age: 43; Male: 0%	Reduction in clinical signs (ISS, VAS, McGill, active range of motion). Secondary: QoL	No significant change in total ISS between groups; withdrawn before significance could be drawn
Van De Vusse, 2004 [32]	CRPS-1, chronic (44 months), pain score >3 VAS	Gabapentin: 600 mg once on days 1–2, 600 mg BID on days 3–4, 600 mg TID on days 5–21	58 total: 29 per group	Mean age: 44; Male: 16%	Pain scores: VAS 0-80 at 3, 5, 8 weeks	Pain relief favored gabapentin in first period (69 to 59 VAS). The effect reduced at week 5, resulting in no overall statistical significance
Sahin, 2006 [33]	CRPS-1, upper extremity, acute	Paracetamol 1500 mg/day vs. nasal salmon calcitonin 200 IU/day + calcium 500 mg/day, with physical therapy/exercise 5/week for 3 weeks	35 total: 17 calcitonin	Mean age: NA; Male: 29%	Pain scores using VAS at 2 months. Secondary: hyperalgesia, allodynia, trophic changes	Early calcitonin showed no additive efficacy vs. paracetamol. No significant secondary outcome differences (P>0.05)
Goebel, 2014 protocol; Goebel, 2017 study [34]	CRPS-1 and CRPS-2, upper and lower extremities, chronic	IVIg 0.5 g/kg infusion. Matching placebo infusions 5 g/100 mL or 10 g/200 mL	108 total: 54 per group	Mean age: 42; Male: 30%	Average 24-h pain intensity over 37 days (days 6-42). Secondary: pain interference (BPI interference subscale), QoL (EuroQoL)	Mean pain scores: placebo 6.9, IVIg 7.2. Adjusted difference 0.27 (95% CI, P=0.30). No significant secondary outcome differences
Groeneweg, 2008 [35]	CRPS-1, lower extremity, cold subtype, chronic	PDE-5 inhibitor 10 mg daily 4 weeks, then 20 mg daily 8 weeks; placebo group followed same titration + physical therapy	24 total: 12 per group	Mean age: 39.8 treatment, 36.5 placebo; Male: 25%	12-week temperature difference in affected limb. Secondary: pain (VAS)	No significant temperature changes. Significant reduction of pain vs. placebo, likely due to increased blood flow
Breuer, 2014 [36]	CRPS-1 and CRPS-2, upper extremity, chronic	IV parecoxib 80 mg/day for 2 days vs. placebo (0.9% NaCl)	20 total: 10 per group	Mean age: 46; Male: 50%	Pain pressure threshold. Secondary: edema, HADS	No significant outcomes at 2 days for primary or secondary measures
Hale, 2023 [37]	CRPS-1 and CRPS-2, chronic	N_^2^_O: 50% N_^2^_O + 50% oxygen vs. air-oxygen 50:50. 3 sessions over 1 week, 2 hours/session. All patients received 2 mg IV midazolam	N_^2^_O: 20; Air-oxygen: 24	Mean age: control 46.3 (male 8%), treatment 44.4 (male 20%)	Primary: pain (PROMIS-29) at 1 week, 1 month. Secondary: physical/mental health Z-scores (PROMIS-29), PGIC	N_^2^_O did not significantly reduce pain. The high baseline pain subgroup saw a modest benefit. No significant improvement in physical/mental health or PGIC
Ratcliffe, 2023 [38]	CRPS, upper and lower extremities, chronic	Soticlestat: Part A 100 mg BID titrated to 300 mg, Part B 200 mg BID adjustable 100–300 mg; placebo control	Part A: 24 total (soticlestat 15, placebo 9); Part B: 18 total (soticlestat 12, placebo 6)	Mean age: 44.5; Male: 29.2%	Primary: mean change 24-h pain score. Secondary: functional, QoL, CSS, PGIC, PROMIS-29	Primary endpoint (15 weeks): no significant difference (-0.34 points, P=0.570). Did not meet clinically meaningful threshold (≥2 points). Functional and QoL: modest improvements; not significant. Biomarker 24HC: Soticlestat ~70% reduction
Arunakul, 2024 [39]	CRPS-1, lower extremity (foot and ankle), acute	Mecobalamin 500 μg or placebo TDS for 3 months; both groups received pregabalin 25 mg up to 300 mg + desensitization therapy	47 total: mecobalamin 24, placebo 23	Mean age: placebo 51.5 (male 13%), treatment 57 (male 29%)	Primary: pain VAS. Secondary: QoL (SF-36), functional outcomes, pregabalin use at 1, 6, 12 months	No significant VAS improvement. Mecobalamin group: significant functional improvement at 3 months; SF-36 scores better at 3 months. Pregabalin total dose and duration were lower in the mecobalamin group at all time points

Due to their known mechanism of action as osteoclast blockers, bisphosphonates have been investigated as an avenue for the treatment of CRPS. Four RCTs have explored their efficacy on pain as a primary outcome. Across these trials, 76 patients were treated with bisphosphonates and then assessed using the visual analogue scale (VAS). Pamidronate was administered in two trials, either as a single infusion [11] or multiple times and compared with steroids [12]. Neridronate was also administered as an intravenous (IV) infusion [13], whereas alendronate was provided orally [14]. On average, there was a follow-up time of six months during which all studies reported statistically significant reductions in pain outcomes (Table 3).

Central sensitization has been proposed as one of the key underlying mechanisms causing CRPS [15]. Consequently, drugs such as Ketamine have recently been evaluated in their efficacy for treatment due to their N-methyl-D-aspartate (NMDA) receptor-antagonizing properties for pain relief [15]. Two studies administered Ketamine intravenously [15,16]. A total of 39 chronic CRPS patients were treated across these studies, both of which reported significant short-term pain relief within weeks 1 and 2. However, these poorer-quality studies did not demonstrate long-standing results (Table 4). Furthermore, topical application of Ketamine seemed to only improve allodynia [17].

Preventing pain transmission through blockage of thoracic or lumbar sympathetic ganglia has been trialed in multiple instances for the treatment of CRPS. An RCT tested whether injection of an anesthetic and steroid solution into the thoracic ganglion would improve brief pain inventory (BPI) scores when compared to injections into the subcutaneous space [18]. At the one-year follow-up, BPI scores were significantly decreased in the treatment cohort (Table 5). Secondary outcomes detected only slight improvements in patient quality of life. Botulinum toxin’s potential to prolong the effects of lumbar sympathetic block has also been investigated [19]. Results showed a statistically significant decrease in pain intensity compared with control, at both one and three months (Table 5). Furthermore, Manjunath et al. sought to compare the efficacy of lumbar sympathetic neurolysis with radiofrequency thermal lumbar sympathectomy in reducing pain for chronic patients. Nine pain outcomes were measured between groups (Table 5). On the whole, both groups experienced significant pain relief, but no statistically significant differences between the two treatment groups were reported [20].

Lidocaine has been combined with different therapeutic agents when investigating ways to treat CRPS. One RCT assessed the effects of an IV block using lidocaine and the non-steroidal anti-inflammatory drug (NSAID) ketorolac [21]. Overall pain score showed that pain relief in the treatment group was greatest at 30 and 120 mg doses, but did not last for longer than one day post-injection. Another study combined lidocaine with clonidine and either systemic or local parecoxib administered intravenously (using a tourniquet), with analgesic effects measured using the VAS and the need for rescue medication during the week following administration. On the whole, the IV parecoxib treatment group required less rescue medication in the second and third weeks compared to the other groups, and VAS scores were lowest for this group by the third week [22]. Local lidocaine injections have also been combined with the antidepressant citalopram. Injections containing 5 cc lidocaine were given every 10 days and combined with oral drops of 40 mg/mL citalopram. An intermediary group received placebo drops with lidocaine injections, and a full control group received placebo injections and placebo drops [23]. Results highlighted that citalopram- and lidocaine-treated patients demonstrated the greatest improvement in impairment level sum scores (ISS) (35 points) compared to lidocaine alone (26 points) and placebo (17 points). Furthermore, subgroup analysis revealed that acute CRPS patients experienced a greater overall reduction in ISS versus chronic CRPS. 

Steroids act to reduce abnormal inflammation in an affected limb. Two RCTs have investigated the efficacy of steroids in 44 patients: in one, a Bier block, where local anesthetic is administered intravenously into an isolated limb, was performed once weekly, and VAS scores were assessed [24]. Although significant during the study, follow-up showed that these effects were no longer significant at the four-week assessment compared with placebo. The other RCT studied the effects of oral prednisolone in post-stroke patients [25]. Results showed that the mean CRPS score decreased from 10.7 to 4.3 within the first month (Table 7). Secondary outcomes such as activities of daily life, measured using the Barthel index, did not show any significant improvement between groups. More recently, a pilot, single-center RCT assessed the efficacy of oral prednisolone in 39 patients with CRPS-1 of the upper extremity [26]. Participants were randomized to receive either 20 mg or 40 mg of prednisolone daily for two weeks, followed by a tapering regimen over the subsequent two weeks. Both dosage groups showed significant improvement in primary outcomes, including pain reduction (VAS) and CRPS scores, as well as in the daily sleep interference scale (DSIS) after one month. In addition, cytokine profiling revealed a decrease in TNF-α and an increase in IL-10 levels, correlating with improved pain and shoulder mobility. No major adverse effects were reported, although dose adjustments to anti-diabetic medication were necessary in a few patients. Importantly, there were no significant differences in outcomes between the two dosage groups. While the findings support the short-term clinical and biochemical benefits of prednisolone in CRPS-1, the study's limited sample size and absence of long-term follow-up highlight the need for further research to determine sustained efficacy and optimal dosing strategies.

Free radical scavengers have been popular in the Netherlands for the treatment of CRPS, primarily based on the theory that following the inciting injury, a disproportionate release of oxygen free radicals occurs [27]. Two RCTs have studied the effects of free radical scavengers, both topically and intravenously. One study found no significant differences in ISS at 17 and 52 weeks when comparing dimethyl sulfoxide (DMSO) cream with a mucolytic drug, N-acetylcysteine (NAC) (Table 7). In addition, upper and lower limb functions were measured using the modified green test and walking stairs questionnaire, respectively. Interestingly, results suggested the cream to be more advantageous in warm CRPS, and NAC to be preferable for cold CRPS function [27]. IV administration of Mannitol has also been explored as a treatment avenue with free radical scavenging properties. Patients were given IV mannitol and compared to saline controls [28]. Subjects were assessed for their VAS scores (Table 7), but no significant differences were found between groups. Furthermore, no encouraging effects were described when assessing hand function. 

Mycophenolate acts to suppress cell-mediated immunity through the inhibition of T-cell and B-cell proliferation [29]. In a trial that treated patients with mycophenolate, four reported significant pain reduction, improved function, and quality of life (Table 7); however, it should be noted that adverse events were experienced by most subjects in the treatment group, including depression in 45% of patients [29]. Lenalidomide, a safer thalidomide derivative with greater anti-inflammatory effects, has also been tested as a treatment. This RCT compared the effects of lenalidomide on pain relief versus placebo [30]. Pain relief greater than 30% on a numeric rating scale (NRS) was not achieved. Activity level rating and other secondary outcomes also showed no favorable outcomes (Table 7). Monoclonal antibodies have also been investigated due to the suggested role cytokines play in the condition. Infliximab was researched as a potential therapy for acute CRPS in six patients, but was discontinued due to a significant number of patients being lost to follow-up [31].

Due to the element of neuropathic pain present in CRPS, anticonvulsants, such as gabapentin, which can modulate neuropathic pain, have been studied. One RCT measured VAS scores in chronic patients and found that short-term analgesic effects were provided, but this did not last the entire treatment phase [32]. 

Calcitonin has been tested in an RCT using nasal salmon calcitonin. VAS diaries revealed no significant improvement at the end of the two-month testing period [33]. Low-dose immunoglobulins have also been given to chronic patients intravenously and compared with placebo infusions; statistical analysis revealed no favorable results for 24-hour pain intensity averages or in quality-of-life tests [34]. 

The phosphodiesterase-5 inhibitor tadalafil is known to increase blood flow and has therefore been assessed in patients with cold-subtype CRPS. Following three months, a 15% decrease in VAS scores was reported [35]. Finally, Breuer et al. investigated IV parecoxib over two days and its effects on pain pressure thresholds. No significant outcomes were reported for this short-term treatment [36]. 

More recent trials have examined novel pharmacological strategies for CRPS with varied outcomes. A double-blind RCT investigated nitrous oxide (N₂O) in 44 adults with chronic CRPS [37]. Participants received either 50% N₂O with oxygen or a placebo for three two-hour sessions over one week. Pain scores were assessed using the Patient-Reported Outcomes Measurement Information System 29-Item Profile (PROMIS-29) at one week and one month. No significant difference was found between groups overall; however, a subgroup with higher baseline pain showed a significant reduction. No serious adverse events occurred, suggesting N₂O may benefit patients with severe pain.

A phase 2a placebo-controlled trial evaluated soticlestat, a cholesterol 24-hydroxylase (CH24H) inhibitor, as adjunctive therapy in 24 patients with chronic CRPS [38]. While soticlestat reduced plasma 24-hydroxycholesterol levels by ~70%, it did not significantly improve 24-hour pain intensity compared to placebo (-0.34 points, P=0.570). Secondary outcomes such as PROMIS-29 scores and responder rates (≥30% pain reduction) also showed no meaningful differences. One patient discontinued due to treatment-related depression. The study was limited by a small sample size and baseline imbalances, and soticlestat was not deemed clinically effective.

A double-blind RCT assessed oral mecobalamin (500 µg TID) in 47 patients with acute CRPS-1 of the foot and ankle [39]. All received standard care, including pregabalin. After three months, the mecobalamin group showed significantly greater improvements in Foot and Ankle Ability Measure (FAAM) scores and in 36-item short-form survey (SF-36) mental health scores. Pain scores were similar between groups, but mecobalamin users required significantly less pregabalin by month four. These findings suggest that mecobalamin may enhance functional recovery and reduce medication reliance in early CRPS-1.

Findings on Pain Reduction for Non-pharmacological Interventions

All non-pharmacological interventions measured pain reduction as a primary outcome. Table 8 (physiotherapy), Table 9 (neuromodulation), and Table 10 (“other mechanisms,” table for unique/novel methods) show non-pharmacological interventions used, with each table grouped by mechanism of action, along with CRPS subtype, study characteristics (demographics and size of patient populations), primary and secondary outcomes, and study results.

**Table 8:** Physiotherapy studies and findings CRPS: complex regional pain syndrome; MT: mirror therapy; VAS: visual analog scale; FIM: functional independence measure; MAS: modified Ashworth scale; NRS: numerical rating scale; SF-12: short-form (12) health survey; MIP: mirror, imagined, and recognition protocol; MAL: motor activity log; PEPT: pain exposure physical therapy; AROM: active range of motion; ISS-RV: impairment level sum score - Raskin and Veltman version; SF-36: short-form (36) health survey; TENS, transcutaneous electrical nerve stimulation; NPS: neuropathic pain scale

Author, year	Diagnosis	Intervention	Patient groups	Demographics	Primary and secondary outcomes	Results
Pervane, 2016 [40]	CRPS-1, upper extremity, dystrophic stage, chronic	The MT group received an additional MT program for 30 min/d vs. control with no MT added. Both groups received a patient-specific conventional stroke rehabilitation program for 4 weeks, 5 d/wk, for 2-4 h/d.	30 total, 15 per group	Mean age: 64; % male: 56	VAS pain score, FIM, MAS spasticity, assessed 1–2 days before and after treatment period	VAS scores in MT: 6 to 3; FIM-motor in MT: 41 to 44. VAS scores in control: 5 to 5; FIM-motor in control: 32 to 39. (P<.001 mt p=0.03 control no significant difference was found for mas scores)
Cacchio, 2009 [41]	CRPS-1, upper extremity, chronic	Four-week conventional stroke rehabilitation program, consisting of five 1-hour sessions/wk: patients observed the reflection of their unaffected upper limb while performing flexions and extensions. Control group performed the same exercise for the same duration, but the mirror was covered.	48 total, 24 per group	Mean age: 58; % male: 45	Pain (2 points on VAS), self-rated allodynia, 6-month follow-up. Secondary: motor function improvement (Wolf Motor Function Test).	VAS score significantly improved in the mirror group. Pre-treatment: 7.6±1.2; post-treatment: 4.3±2.5; follow-up: 4.7±2.6. Motor function scores: pre-treatment: 3.5±1.2; post-treatment: 1.5±0.7; follow-up: 1.9±1.2 (P<0.001).
Ozdemir, 2024 [42]	CRPS-1, upper extremity, acute	Both groups received 20 sessions (4 weeks) of physical therapy (including exercises, TENS). The treatment group also received an additional 30 minutes of MT per session.	40 total, 20 per group	Control: mean age 50.85 years, % male: 25. Mirror: mean age 52.55 years, % male: 30	Primary: pain on NRS. Secondary: grip strength, pinch strength, wrist dexterity, and function assessed at baseline (4 weeks) and at 8-week follow-up.	Both groups showed statistically significant improvements in pain, grip/pinch strength, wrist circumference, dexterity, and hand function. No statistically significant differences between the mirror and control groups in change scores across all outcome measures (P>0.05).
Machac, 2024 [43]	CRPS-1, upper extremity, acute and chronic	MT for 10 minutes daily over 6 weeks. Exercises: pronation, supination, clapping, fist motions, finger walking. First 5 minutes: only the unaffected hand moved. Last 5 minutes: slight movement allowed in the affected hand. Participants observed a mirror reflection to create a visual illusion of affected hand movement.	28 total, 14 per group	Group A: mean age 57 years, % male: 8. Group B: mean age 55.3 years, % male: 21	Primary: pain using VAS, follow-up 30 days post-intervention via 1–10 verbal numerical rating, and hand function. Secondary: health-related quality of life.	Significant improvements observed in pain reduction (rest and movement pain) post-MT, with lasting benefits at 30-day follow-up. Hand function improvements: increased grip strength (+25.5%, P=0.02), wrist flexion (dorsal +26.3%, P=0.02), reduced finger-to-palm distance (P=0.04). Quality of life improved by 13.3% (P=0.04).
Saxena, 2022 [44]	CRPS-1, upper extremity, acute	Both groups: oral pregabalin (75 mg twice daily for 12 weeks) and scrubbing rehabilitation technique (15 minutes, twice daily). MT group: MT with exercises for the unaffected upper limb while observing reflection in the mirror (opening/closing fists, wrist flexion/extension, forearm supination/pronation, elbow flexion/extension, shoulder flexion/extension), each exercise performed 30 times per set, repeated thrice.	40 total, 20 per group	Control: mean age 48.3 years, % male: 50. Treatment: mean age 49.85, % male: 55	Primary: pain relief (NPSI and NRS). Secondary: quality of life (SF-12). Timepoints: weeks 2, 4, 6, 8, and 12.	Significant reduction in pain intensity: lower NRS pain scores, NPSI allodynia, and NPSI burning scores, with improvements in motor function (modified MAL scores) and quality of life in the MT group vs. control. This approach led to downregulation of mTORC1 and IL-6 mRNA expression, indicating reduced chronic inflammation contributing to pain relief (P<0.05).
Moseley, 2004 [45]	CRPS-1, upper extremity, chronic	Six-week MIP treatment: MT, imagined hand movements, and right/left hand recognition vs. control (ongoing medical management). If the hypothesis is supported at 12 weeks, the control crossed over to MIP.	13 total, 7 MIP, 6 control	Mean age: 38; % male: 30.8	Reduction in neuropathic pain, on a scale of 0=100. No longer fulfilling criteria for CRPS-1.	Six weeks post-MIP: 50% no longer fulfilled diagnostic criteria for CRPS-1 (P=0.01). Number needed to treat for 50% reduction in pain: 3.
Moseley, 2005 [46]	CRPS-1, upper extremity, chronic	Group 1: Rec-Im-Mir (recognition of hand laterality, imagined hand movements, mirror movements). Group 2: Im-Rec-Im. Group 3: Rec-Mir-Rec. Optional physiotherapy during 12-week follow-up.	20 total (G1=7, G2=6, G3=7)	Mean age: 36; % male: 30	2-, 4-, 6-week assessment: neuropathic pain (NPS scale). Overall function measured using the task-specific scale and NRS.	At 6 weeks: the greatest NPS decrease in group 1 (approx. 50 to 30) (P<0.05). Treatment effective only in a specific order. Group 1 also had the most functional improvement at 12 weeks (approx. 5 to 25) (P<0.05).
Barnhoorn, 2012 (protocol), Barnhoorn, 2015 (study) [47]	CRPS-1, upper and lower extremities, acute and chronic	Experimental: PEPT, max. 5 sessions (40 min each), no analgesics. Control: analgesics, N-acetylcysteine, calcium channel blocker, ketanserin, and dimethyl sulfoxide for local skin application.	56 total, 28 per group	Mean age: 44.5; % male: 20	Impairment level sum score (ISS), VAS, AROM, and temperature (3, 6, 9 months). Secondary: SF-36 for quality of life.	PEPT had greater AROM vs. control (between-group difference 0.51, 95% CI 0.07-0.94; P=0.02). Clinically relevant ISS-RV decrease (6.7 points PEPT, 6.2 points control), but between-group difference not significant (0.96, 95% CI -1.56 to 3.48).

**Table 9:** Neuromodulation studies and findings CRPS: complex regional pain syndrome; SCS: spinal cord stimulation; PT: physiotherapy; VAS: visual analog scale; QoL: quality of life; MPQ-DLV: McGill pain questionnaire - Dutch language version; DRG: dorsal root ganglion; SF-36: short-form (36) health survey; TENS: transcutaneous electrical nerve stimulation; LANSS: Leeds assessment of neuropathic symptoms and signs; DN4: douleur neuropathique 4; DHI: Duruoz hand index; PEMF: pulsed electromagnetic field; NRS: numerical rating scale

Author, year	Diagnosis	Intervention	Patient groups	Demographics	Primary and secondary outcomes	Results
Kemler, 2008 [48]	CRPS-1 restricted to one extremity, chronic	SCS + PT group or a PT alone group; 7-day test, 50% improvement required for SCS device permanent implantation; 5-year follow-up	54 total: 36 SCS, 18 PT alone	Mean age: NA; % male: NA	5-year follow-up pain - VAS, McGill pain questionnaire; secondary: asked whether they would repeat treatment (based on global perceived effect) and quality of life using EuroQoL	SCS did not lead to statistically significant improvement in pain at 5 years. No significant improvement in QoL score. Nevertheless, 95% of patients said yes to repeating treatment.
Kriek, 2015 protocol; Kriek, 2017 study [49]	CRPS, one extremity only, chronic	Five different SCS frequencies: standard 40 Hz, 500 Hz, 1200 Hz, burst, and placebo stimulation over a 10-week crossover period; placebo: 100 Hz standard stimulation	40 total	Mean age: 42; % male: 4%; % white: 97%	Primary: pain reduction and patient satisfaction measured with the MPQ-DLV and VAS	VAS scores were lowest for standard (39.83) vs. placebo (63.74) (p<0.001). McGill scores for maximum pain were significantly lower for standard stimulation (6.31), 500 Hz (6.86), and 1200 Hz (6.52), compared with placebo (8.35) (p=0.001). Burst stimulation showed no significant difference.
Deer, 2017 [50]	CRPS-1, lower extremity, chronic	DRG stimulation (DRG group) or traditional SCS (SCS group) in a 1:1 ratio. Leads were placed between levels T10 to S2 depending on where subjects had the most pain	152 total: 76 DRG, 76 SCS	Mean age: 52; % male: 49%	3 months safety and efficacy; 12 months treatment success defined as a 50% reduction in VAS; SF-36	DRG stimulation had a higher rate of treatment success (81.2%) compared with the treatment success rate for traditional SCS (55.7%) (p=0.001). Pain relief and greater quality of life in the DRG group persisted for a 12-month follow-up.
Bilgili, 2016 [51]	CRPS-1, upper extremities, acute and chronic	Group 1: TENS + contrast bath + whirlpool bath + exercise program; group 2: sham TENS + contrast bath + whirlpool bath + exercise program. Conventional TENS: 100 Hz, pulse duration 50-100 ms	30 total: 15 in each group	Mean age: 47; % male: 46%	VAS to assess spontaneous pain; the LANSS; DN4; functional capacity using DHI	Mean change in group 1 VAS: -33.20; mean change in group 2 VAS: -12.07. Mean change in group 1 LANSS: -4.60; mean change in group 2 LANSS: -2.07. Mean change in group 1 DN4: -2.20; mean change in group 2 DN4: -1.53 (p<0.05). No significant difference observed in the DHI score.
Comertoglu, 2022 [52]	CRPS-1, upper extremity, acute	Control: bath therapy, hot pack, TENS, exercises, and desensitization. PEMF: same conventional rehabilitation plus PEMF therapy applied to the affected hand and wrist using a PMT Quattro Pro device, with 3.2 mT intensity, 8 Hz frequency, for 20 minutes/day	32 total: 16 PEMF, 16 control	Mean age: 50; % male: 50%	Primary: NRS to assess pain scores; secondary: grip strength, pinch strength, edema of hand, dexterity	No significant difference between groups in pain reduction. Both groups showed significant improvements from baseline to the 1-month follow-up in pain, grip and pinch strength, hand edema (circumferential and ultrasonographic), hand dexterity, and hand activities.

**Table 10:** Other non-pharmacological studies and findings CRPS: complex regional pain syndrome; FIM: functional independence measure; VAS: visual analog scale; BPD: body perception disturbance; NRS: numerical rating scale; ES: effect size; OS: occlusal splint; SF-36: short-form (36) health survey

Author, year	Diagnosis	Intervention	Patient groups	Demographics	Primary and secondary outcomes	Results
Ozcan, 2019 [53]	CRPS-1, subacute, post-stroke	Experimental group received 15 sessions of additional fluidotherapy added to a conventional stroke rehabilitation program (40°C, 20 minutes in continuous mode, 5 sessions per week); no sham for control	32 total, 16 per group	Mean age: 64; % male: 66%	Brunnstrom recovery stages of the arm and hand for motor recovery; FIM; VAS for pain severity; PainDETECT questionnaire for severity of neuropathic pain	Only PainDETECT scores were greater in the fluidotherapy group (score difference 4) than in the control group (score difference 2) (p<0.05).
Lewis, 2021 [54]	CRPS, upper limb, chronic, with BPD	One-minute visual image digitally altered according to the patient’s description using the MIRAGE system. “How satisfied are you with the hand as you see it?” was rated on a 7-point Likert scale. Control: procedure and duration for non-manipulation were the same, but the image was not altered.	45 total, 23 experimental, 22 control	Mean age: 52; % male: 35%	BPD scale to measure changes in body perception of the affected limb; current pain intensity rated verbally on an 11-point NRS	BPD (mean -6±7.9, p=0.036, effect size (ES)=0.6) and pain intensity (mean -0.43±1.3, p=0.047, ES=0.5) were reduced in 23 participants after a single exposure compared with controls (n=22). At follow-up, the subgroup (experimental n=21; control n=18) showed sustained pain reduction only (p=0.037, ±1.9, ES=0.7), with an overall 1.2 decrease on the 11-point scale.
Fischer, 2008 [55]	CRPS-1, upper and lower extremities, chronic (35-41 months duration)	Occlusal splint used at night and for 3 hours during the day for a total of 7 weeks; compliance assessed with a self-questionnaire	20 total, 10 per group	Mean age: 43 in controls, 51 in OS; % male: 20%	Self-reported pain using 0-10 scales; SF-36; assessed at 7 weeks	No significant differences between the two groups (p=0.720). No significant differences in SF-36 performance at baseline (p=0.292).

Mirror therapy (MT) can manipulate the functions of motor and sensory cortices in post-stroke neurorehabilitation, particularly in the treatment of phantom limb pain [40]. Two RCTs analyzed the efficacy of MT on VAS scores in a total of 39 patients with chronic CRPS [40,41]. Both studies demonstrated significant improvements (Table 8), with findings from Cacchio et al. lasting until the six-month follow-up, while Pervane Vural et al. had no follow-up period [40,41]. Additionally, Cacchio et al. found that motor function showed statistically significant differences between treatment and control groups [41]. More recent studies further support the utility of MT in CRPS treatment, although with nuanced outcomes. 

A prospective, single-blind RCT involving 40 patients with acute post-traumatic CRPS-1 of the hand compared conventional physical therapy with and without the addition of MT. Both groups showed significant within-group improvements in pain, strength, dexterity, wrist circumference, and function. However, no statistically significant between-group differences were found in outcome measures at any time point, although the MT group demonstrated non-significant trends toward improved health-related quality of life in pain, sleep, and emotional domains [42]. In contrast, a half cross-over trial conducted at University Hospital Motol with 28 patients diagnosed with unilateral upper extremity CRPS I found significant improvements following MT. Pain scores at rest and during movement decreased, and gains in hand function, including grip strength, wrist flexion, and reduced finger-to-palm distance, were observed. Quality of life improved significantly (+13.3%, P=0.04), with effects maintained at 30-day follow-up. MT also appeared to mitigate neurophysiological deficits such as distorted body image and neglect-like symptoms. The study emphasised the benefits of early intervention and highlighted MT's cost-effectiveness compared to pharmacological and electrotherapy options [43].

Moreover, MT combined with pregabalin has shown synergistic effects in CRPS-1 management. In a recent trial, patients receiving both treatments exhibited significantly greater reductions in pain using NRS, neuropathic pain symptom inventory (NPSI), allodynia, and burning scores, alongside improved motor function and quality of life, compared to those receiving pregabalin alone. Molecular analyses revealed a downregulation of mTORC1 and IL-6 mRNA expression, suggesting that MT may help reduce chronic inflammation, a key factor in CRPS pathogenesis. This study aligns with previous research linking MT to gene modulation and cytokine regulation [44]. Collectively, these findings reinforce MT as a viable adjunctive or first-line intervention for CRPS, with demonstrated benefits in pain reduction, motor recovery, and quality of life. However, limitations such as small sample sizes, short follow-up periods, and limited use of neuroimaging suggest the need for larger, multicentric RCTs with advanced methodologies to confirm these promising outcomes.

Motor imagery programs (MIP) involve thinking about performing movements, without moving the affected limb. Two RCTs have investigated this mode of treatment and whether the order of components involved plays a role in the results obtained. The first study showed that neuropathic pain was reduced so significantly that after six weeks, half of the participants were no longer classified as having CRPS-1 [45]. A follow-up trial showed that treatment group 1, starting with the recognition of the laterality component, followed by imagined movements and then by MT, had the largest decrease in the neuropathic pain scale (NPS) compared to other groups receiving alternative combinations (Table 8). Overall function was also measured, and the same group demonstrated the most improvement [46]. 

Pain exposure physical therapy (PEPT) encourages patients to partake in daily activities using their affected limb, regardless of pain experienced, and without analgesic relief. A single RCT studied 56 subjects, of whom half received various PEPT sessions, depending on patient need [47]. When compared to conventional physiotherapy, PEPT failed to elicit any significant improvement in overall ISS, although patients did have better active range of motion (AROM). Other assessments measuring quality of life, such as the short-form survey 36 (SF-36), also failed to exhibit any inter-group significance (Table 8). However, it was reported that more sessions were required in the conventional therapy group versus a maximum of five sessions in the PEPT group.

Spinal cord stimulation (SCS) requires implantation of a device subcutaneously, which allows low levels of electricity to stimulate the spinal cord and, in doing so, inhibit chronic pain. Two RCTs have investigated SCS in CRPS; the first examined whether SCS provided lasting pain relief (Table 9). The study showed significant analgesic effects remained until the third year and that 19/20 patients said they would repeat the treatment [48]. Secondary outcomes showed no improvement in the treatment group compared to the control. Furthermore, 42% of patients experienced numerous complications and had to undergo re-operation, which is an established feature of SCS treatment. The second RCT investigated whether different frequencies of SCS provided better analgesic effects in chronic patients. All five settings demonstrated significant pain reduction compared to placebo (Table 9). Interestingly, patients could also decide which setting they preferred, and almost half (48%) chose standard (40Hz). Nevertheless, the lowest pain scores were reported at alternative frequencies in 72% of patients. These findings suggest that SCS therapies may need to be personalized to patients’ preferences [49]. SCS has also been compared with dorsal root ganglion (DRG) stimulation [50]. At three months, a 50% improvement in VAS scores was observed in 25.5% more patients in the DRG arm compared with SCS. Secondary outcomes showed greater benefits from DRG than SCS, which were maintained at follow-up.

Transcutaneous electrical nerve stimulation (TENS) has also been trialed alongside standard physical therapy. An RCT investigated the effects of conventional TENS therapy versus placebo sham TENS over 15 sessions [51]. Spontaneous pain and neuropathic pain were assessed as primary outcomes, with functional capacity as a secondary outcome (Table 9). Statistically significant improvements were reported in both groups, but neuropathic pain scores and functional capacity were substantially better in the conventional TENS treatment arm [51].

In a related area of adjunctive therapy, pulsed electromagnetic field (PEMF) therapy has been evaluated for its potential benefit in CRPS-1 management. A single-blind, RCT investigated the effectiveness of PEMF in conjunction with conventional rehabilitation in 32 patients with CRPS-1 of the hand. Participants were randomized into two groups: one receiving standard rehabilitation (including contrast baths, hot packs, TENS, desensitization, and exercises) and the other receiving the same program with the addition of PEMF therapy (3.2 mT intensity, 8 Hz frequency). Outcome measures included pain severity, grip and pinch strength, hand edema (both circumferential and ultrasonographic), hand dexterity (Moberg pick-up test), and daily functional ability (Duruöz hand index). Although both groups showed significant improvements across all outcomes after one month, no statistically significant differences were found between them (Table 9). These findings suggest that the addition of PEMF did not confer additional clinical benefit over conventional rehabilitation alone. The study underscores the need for further research using sham-controlled designs and varied PEMF parameters to better elucidate its role in CRPS treatment [52].

Fluidotherapy, utilizing mechanical and thermal stimuli to provide a therapeutic effect, has been explored in post-stroke CRPS patients. Results showed no beneficial effects according to VAS scores and functional independence in the treatment group [53]. Virtual reality (VR) has been used to change the way patients visualize their CRPS-affected hands by digitally altering the image to match their ideal descriptions. Pain score results suggested a sustained analgesic effect (Table 10) with repeated VR exposure [54]. Occlusal splints have also been trialed, but after seven weeks, no significant improvement was reported [55].

Adverse Effects and Safety Profile

Serious adverse effects (SAEs) were measured across many of the RCTs, with some instances resulting in patients dropping out of studies altogether. SAEs were aggregated across intervention groups in Table 11 in order to highlight interventions that potentially present with increased risk to the CRPS patient. Where significant SAEs were recorded, including dropouts, these individual RCTs are shown in Table 12.

**Table 11:** Aggregate of serious adverse events and patient dropouts across all RCTs RCTs, randomized controlled trials

Intervention group	Number of studies with at least one SAE	Number of studies with at least one dropout
Bisphosphonates (n=4)	0	2 (50%)
Ketamine	0	0
Sympathetic blocks	0	0
Other pharmacological interventions (n=16)	7 (43.75%)	4 (25%)
Physiotherapy	0	0
Neuromodulation (n=5)	2 (40%)	0
Other non-pharmacological interventions	0	0

**Table 12:** Individual studies presenting with serious adverse events and/or patient dropouts due to these events CRPS: complex regional pain syndrome; DMSO: dimethyl sulfoxide; NAC: N-acetylcysteine; SCS: spinal cord stimulation; PT: physiotherapy; DRG: dorsal root ganglion; SAE: serious adverse event; TEAE: treatment-emergent adverse event

Author, year	Serious adverse events	Patient dropouts
Varenna, 2013 [13]	Treatment group: 21 patients reported at least one adverse event (12 were drug-related, mainly polyarthralgia). Placebo group: 12 patients reported at least one adverse event (5 musculoskeletal disorders, 1 fever). Open phase: 1 patient dropped out due to an adverse event, and 14 patients reported drug-related adverse events.	Treatment group: 1 patient dropped out; Placebo group: 1 patient dropped out; Open phase: 1 patient dropped out
Manicourt, 2004 [14]	Treatment group: upper gastrointestinal intolerance. Placebo group: no serious adverse events, only minor ones (nausea, dizziness)	Treatment group: 1 patient dropped out
Taskaynatan, 2004 [24]	Treatment group: syncope and subjective palpitation. Placebo group: subjective palpitation	Treatment group: 2 patients dropped out; Placebo group: 1 patient dropped out
Perez, 2003 [27]	DMSO group: severe skin reactions. NAC group: severe stomach complaints	DMSO group: 3 patients dropped out; NAC group: 5 patients dropped out
Goebel, 2018 [29]	Four adverse events classed as severe (nausea, blackout followed by fall with facial contusion, skin itchiness). Two events after physical accidents in the same patient were classed as serious and judged as not drug-related	None
Manning, 2014 [30]	Treatment group: 4 patients reported at least 1 serious adverse event (decreased potassium, pain, joint sprain, angioneurotic edema). Cumulative data showed that treatment-related adverse events led to discontinuation in 29 patients. Placebo group: 6 patients reported at least 1 serious adverse event (increased blood pressure, neuropathic pain, vomiting, tremor, Clostridium colitis, respiratory failure)	Treatment group: 18 patients discontinued treatment; Placebo group: 5 patients discontinued treatment; cumulative data showed treatment-related adverse events led to discontinuation in 29 patients who crossed over following placebo
Dirck, 2013 [31]	Treatment group: none. Placebo group: 1 serious adverse event reported, unrelated to treatment	None
Goebel, 2017 [34]	Treatment group: 1 severe adverse event (severe headaches) requiring hospitalization. Placebo group: 1 severe adverse event (headaches, vomiting) requiring hospitalization	Treatment group: 3 patients withdrew; placebo group: 3 patients withdrew
Ratcliffe, 2023 [38]	One serious psychiatric treatment-emergent adverse event (depression/suicidal ideation) possibly related to soticlestat. Other serious treatment-emergent adverse events (e.g., cholecystitis) were unrelated to treatment	None
Kemler, 2008 [48]	Treatment group: 3 explanations of the system	None
Deer, 2017 [50]	A total of 21 serious adverse events occurred in 19 subjects (8 DRG subjects and 11 SCS subjects). Two of the SAEs were adjudicated as definitely related to the implant procedure	None

Discussion

Summary of Key Findings

This review synthesizes the available evidence from RCTs examining diverse interventions for the treatment of CRPS, highlighting the heterogeneity of mechanisms targeted and outcomes reported. The findings reflect a multifaceted therapeutic landscape, where both pharmacological and non-pharmacological strategies demonstrate varying degrees of efficacy.

Bisphosphonates (pamidronate, neridronate, and alendronate) consistently demonstrated significant pain relief in CRPS [11-14], particularly when administered intravenously [11-13]. However, variations in dosing and small sample sizes necessitate further standardization. Ketamine showed short-term analgesic effects in chronic CRPS, primarily via IV administration [15,16], while topical applications offered limited benefit [17]. Combinations of lidocaine with agents such as citalopram or parecoxib produced transient pain reduction [22,23], especially in acute CRPS cases [23], though long-term efficacy remains unclear [21-23]. Corticosteroids provided short-term symptom relief, possibly through anti-inflammatory cytokine modulation, but functional improvements were inconsistent [24-26]. Other pharmacologic agents, including gabapentin [32], calcitonin [33], free radical scavengers [27], and immunosuppressants [31,34], yielded mixed or minimal benefits, often accompanied by adverse events [27,29,30,34,38].

MT emerged as a promising non-invasive approach, particularly effective in early-stage or post-traumatic CRPS, improving pain, motor function, and quality of life [40-46]. MT combined with pregabalin showed synergistic effects and was associated with biological changes such as reduced inflammation [44]. MIP, structured progressively from laterality recognition to imagined movement and MT, enhanced neuroplasticity and reduced neuropathic pain [45,46]. PEPT was comparable to conventional therapy in outcomes but required fewer sessions, suggesting cost-effectiveness [47]. Neurostimulation techniques, including DRG and SCS, provided substantial pain relief [48-52], with DRG stimulation offering greater precision and patient satisfaction [50]. However, SCS was linked to a higher incidence (Table 12) of complications [48-50]. TENS improved neuropathic pain and function [51,52], while PEMF therapy did not outperform standard rehabilitation [52]. Other novel non-pharmacological interventions require further validation [53-55].

Among the included RCTs, 31.8% were judged to have low risk of bias, 40.9% had some concerns, and 27.3% were rated as high risk (Figure 3); high risk of bias was primarily due to issues with randomization, blinding, and incomplete outcome data, which weaken the confidence in pooled results. The heterogeneity in trial design, small sample sizes, inconsistent outcome measures, and lack of adequate reporting on CRPS subtypes, particularly acute and type 2 CRPS, limited the reliability and generalizability of findings. Differences in patient populations, intervention protocols, and pain outcome measures also introduced variability that complicated direct comparisons and synthesis. These methodological limitations underscore the urgent need for well-designed, standardized trials with clear stratification to improve evidence quality.

Comparison With Existing Literature

This review largely corroborates earlier systematic reviews and meta-analyses that identify bisphosphonates [56] and MT [57] as consistently effective interventions for CRPS pain reduction. The analgesic effects of IV bisphosphonates, such as pamidronate [11,12] and neridronate [13], and the functional improvements linked to MT [40-46], echo prior conclusions regarding their central role in CRPS management. Similarly, the transient analgesic benefit of ketamine observed aligns with earlier evidence emphasizing its short-term utility rather than sustained relief [56]. However, our review expands upon previous work by incorporating recent trials on neurostimulation methods like DRG stimulation, suggesting improved precision and patient satisfaction compared to SCS [50]. This added focus on neuromodulatory techniques and emerging immunomodulatory effects of physiotherapy contributes fresh perspectives to the field, highlighting evolving therapeutic avenues not yet fully captured in prior reviews.

Clinical Treatment Recommendations

Current CRPS management typically involves a combination of physical therapy, pharmacological treatment, and psychological support. Early intervention is crucial to improve outcomes [58]. Given the evidence outlined in this review, IV bisphosphonates should be considered a frontline pharmacological treatment for pain relief in CRPS, especially in medium-term management [11-13]. MT emerges as a highly recommended non-pharmacological intervention, particularly in early or post-traumatic CRPS, due to its favorable safety profile and demonstrated benefits on pain, motor function, and possibly inflammatory processes [40-46]. Ketamine may be reserved for short-term symptom control in refractory or chronic cases but requires careful monitoring due to transient effects and potential side effects [15-17]. Neurostimulation therapies, including DRG and SCS, offer significant analgesia but demand individualized patient selection and comprehensive counseling on risks, given the high complication rates and number of SAEs associated with SCS [48-52]. Corticosteroids may have a role in acute phases but should be used cautiously due to the limited duration of effect and uncertain functional benefits [24-26]. Multi-modal approaches combining pharmacological agents with physiotherapy and neuromodulation are likely optimal, emphasizing personalized treatment plans tailored to disease stage and patient characteristics. However, definitive treatment pathways remain constrained by limited long-term data and trial heterogeneity.

Review Limitations

The limitations in this review are mainly characterized by its inability to perform a meta-analysis or subgroup analysis. A meta-analysis was planned when data for the same primary outcome, treatment route, and patient population were available from three or more studies; otherwise, a narrative synthesis would be performed. Due to clinical and methodological heterogeneity across RCTs, along with a significant presence of low- and high-concern RoB-2 scores for an overall low number of studies per intervention type, a narrative synthesis was conducted to avoid a potentially misleading summary effect from a meta-analysis. Two subgroup analyses were pre-specified in the review protocol, aiming to separately evaluate outcomes based on CRPS acuity (acute versus chronic) and CRPS type (type 1 versus type 2). These subgroup assessments aimed to identify whether differences in acuity or CRPS type influenced the magnitude or direction of treatment effects. However, due to the limited number of studies involving acute CRPS and the absence of studies reporting on CRPS type 2, conducting these planned subgroup analyses was not feasible. Future research should prioritize clearer stratification and comprehensive reporting of CRPS subtypes, particularly focusing on acute CRPS and type 2 CRPS populations, to better inform treatment recommendations tailored to these groups and to rule out biased treatment of CRPS toward chronic/type 1 CRPS patients.

Most studies explicitly addressed CRPS-1, with limited representation of type 2 or mixed types, while also leaning strongly toward chronic CRPS patients. Future reviews will require the generation of new RCTs with clearer stratification and comprehensive coverage of both CRPS subtypes, particularly ensuring more equitable inclusion of acute CRPS and type 2 CRPS populations, to better inform treatment recommendations tailored to these groups and to prevent biasing CRPS treatment toward chronic/type 1 CRPS patients.

Publication bias is also a concern, as studies reporting positive outcomes tend to be preferentially published, skewing the evidence base. We attempted to mitigate this by including reports and exposing any publication bias via a funnel plot, highlighting that residual bias cannot be excluded. Finally, language restrictions to English publications and the exclusion of non-RCT designs may have omitted relevant evidence required to build a comprehensive treatment picture for CRPS.

Future Directions

This review underscores the complexity of CRPS and the variable effectiveness of current treatments. While bisphosphonates, MT, ketamine, and electrical neuromodulation show the most consistent promise, their clinical application is limited by methodological inconsistencies, small sample sizes, and a lack of stratified data by CRPS subtype. For non-pharmacological interventions, provider effect can also impact the primary outcomes, potentially resulting in inconsistent results. In future studies, a shift toward individualized, multimodal strategies grounded in subtype-specific research is essential to improving outcomes in this challenging condition. Future research should ideally prioritize large, multicenter RCTs with stratification based on CRPS subtype (e.g., type I vs. II, acute vs. chronic), as acute CRPS and type 2 cases were markedly underrepresented or absent. This skew limits the external validity of findings across the heterogeneous CRPS spectrum. Furthermore, biomarker profiling and neuroimaging could be explored in further depth to better identify responders and elucidate mechanisms of action. Integrating behavioral, pharmacological, and neuro-modulatory approaches in a patient-tailored framework may also offer the most effective pathway for managing this complex and often debilitating condition.

## Conclusions

This comprehensive review of RCTs underscores the complex and multifactorial nature of CRPS, both in its pathophysiology and treatment response. Bisphosphonates, MT, MIP, and neurostimulation techniques demonstrate consistent benefits in selected patient populations. The variability in treatment outcomes highlights the importance of tailoring interventions to CRPS subtype, disease stage, and individual symptom profiles. Equally, the inconsistent methodological quality, small sample sizes, and lack of standardized outcome measures across trials limit the strength of current evidence and hinder clinical decision-making. 

Moving forward, a personalized approach that combines mechanism-based therapies, robust phenotyping, and personalized rehabilitation strategies holds the greatest promise for improving outcomes in CRPS. Well-powered, stratified RCTs with long-term follow-up are urgently needed to refine treatment pathways and guide evidence-based care for this challenging condition.
